# Supplementary material for: Genomic analysis of cardiac surgery-associated Mycobacterium chimaera infections in Italy
Source: PLoS One. 2020 Sep 25;15(9):e0239273. doi: 10.1371/journal.pone.0239273 (PMC7518601; doi:10.1371/journal.pone.0239273)
Supplement: Attachment — Submitted filename: Ghodousi et al. rebutal letter-PLOS ONE.docx [file pone.0239273.s002.docx]

13^th^ of August 2020

To the Editor,

On behalf of all the authors I would like to thank the editor and the reviewers for careful reading and specific and helpful comments to our manuscript. We have revised the manuscript according to all the comments and suggestions provided.

We think that the paper is now substantially improved and we are submitting our revised manuscript for your consideration after all Authors have approved the revision.

The name of the manuscript in word format is: “Ghodousi et al_Revised Article-File.docx”

The title of the manuscript is: “Genomic Analysis of Cardiac Surgery-Associated Mycobacterium chimaera Infections in Italy”

Manuscript no: PONE-D-20-09309

I hope that you find this new version of the manuscript suitable for publication.

Sincerely,

Daniela M.Cirillo, MD, PhD

**Response to reviewers:**

Editor# Line 64: Please clarify whether "10-fold coverage" means coverage by at least 10 reads or something else.

Thanks for the comment. We have re-wrote the phrases to clarify “10-fold coverage”.

Line 64: Please provide more detail of the settings used for variant calling in samtools and GATK.

Thanks for the comment. More details of the setting for variant calling were added to the manuscript.

Line 69: Typo: should be "GrapeTree"

It is now corrected.

Line 70: Minimum 2-fold (or read?) coverage seems like it would identify many sequencing errors as false-positive variants. What steps were taken to ensure this was not the case?

We’d like to thank for this comment. In this case we have used the parameters in which the majority allele other than wild type is considered per position. For each isolate, we calculated the mean allele frequency for each set of SNP alleles, setting the following thresholds: minimum mapping quality of 20, minimum base quality at a position of 20, minimum read depth at a position of 2X, maximum strand bias for a position 90% in order to distinguish the real genomic variants from possible sequencing errors.

Line 88: The term "very similar" should be more specifically defined or quantified.

Thanks for the comment, the mentioned term was defined more specifically in the text.

Line 100: Should define the abbreviations used in the figure (i.e. "P. data") in the figure legend for clarity.

The figure legend has been corrected.

Line 113: Describe how ANI was determined

The calculation of ANI has been described in Materials & Methods section.

Line 114: Clustal X and SplitsTree4 should be referenced and discussed in the materials and methods section.

Thanks for the comment. Clustal X and SplitsTree4 have been referenced and discussed in the materials and methods section.

Line 131: A supplemental table listing the isolates sequenced, their group assignments, and SRA accession numbers should be added.

Thank you for this comment. A supplementary table listing the isolates sequenced, their group assignment, and SRA accession numbers has been added and submitted with the revised manuscript.

Line 166: The PLoS One editorial office will be following up with you re: further clarification of the Ethics Statement.

Reviewer #1: Excellent and concise analysis of the M.chimaera associated with HCU in the authors' country (Italy). The statistical details of the analyses were a bit low. Why use the type strain DSM 44623T versus ZUERICH1 or CDC 2015-22-71 as references for the mapping of the reads? The application of published SNP thresholds is appropriate, but the distribution and depiction of the SNP variation observed in HCU isolates from Italy is absent. Percentages of HCU clone versus non-related genotypes were absent from the text as well. Just a few more statistical numbers included in the report would be appropriate. A table or supplementary table of isolates, name, origin, and SRA or biosample number should be included in Supplementary materials for readers. Authors omitted citing the two HCU genomic analyses from the US CDC/National Jewish Health and the genomic analyses of Australian/New Zealand isolates.

Response:

We would like to thank the reviewer for the comment. In order to analyze the WGS data we have used the *M. chimaera DSM 44623* and also *M. chimaera strain Zuerich1* as reference genome and we didn’t find significant differences for phylogenetic analysis. However, genome size of *M. chimaera DSM 44623* and *strain Zuerich1* are different (5,865,644 vs 6,175,731 bp, respectively). For genotyping we extracted a set of variants specific for (sub)groups of isolates defined by the phylogenetic analysis with respect to the *M. chimaera DSM 44623* genome and its annotation with reference to van Ingen et al. *Lancet Infect Dis* 2017;17: 1033–41.

Moreover, a more detailed statistic of HCU and clinical isolates was included the result section.

A supplementary table of isolates, name, origin, and SRA accession number has been included in Supplementary materials.

Finally, the two mentioned papers were cited in the manuscript and included in the references.
